# Chromatin Biology Impacts Adaptive Evolution of Filamentous Plant Pathogens

**DOI:** 10.1371/journal.ppat.1005920

**Published:** 2016-11-03

**Authors:** Michael F. Seidl, David E. Cook, Bart P. H. J. Thomma

**Affiliations:** Laboratory of Phytopathology, Wageningen University, The Netherlands; THE SAINSBURY LABORATORY, UNITED KINGDOM

## Introduction

An organism’s adaptation to changing environments is fueled by its genetic variability, which is established by mechanisms ranging from single-nucleotide polymorphisms to large-scale structural variations, all of which affect chromosomal shape, organization, and gene content [1]. These processes are particularly relevant for pathogens that must respond to continual selection pressure arising from host immune systems that evolved to detect the presence or activity of potential microbial pathogens through a variety of invasion patterns [2]. In their adaptive response, pathogens evolve strategies, often involving secreted effector molecules, to overcome host immunity and support host colonization [3]. Thus, it can be anticipated that this coevolutionary arms race leads to highly specific interactions between adapted pathogens and their specific hosts. Paradoxically, particular pathogens successfully colonize a broad range of hosts, yet how such pathogens cope in arms races with such a diversity of hosts remains unknown.

### A structured genome drives adaptive evolution

It has been proposed that filamentous fungal and oomycete plant pathogens often evolved structured genomes with two distinct types of genomic regions: (1) gene-rich regions containing slowly evolving genes that mediate general physiology and (2) gene-poor regions that are dynamic and enriched for repetitive DNA such as transposable elements (TEs) and virulence-related genes that often display signs of accelerated evolution [4,5]. Extensive structural variation often occurs in these fast-evolving regions, leading to translocation, duplication, or loss of genetic material [1,4,5]. The highly dynamic regions can either be embedded within the core chromosomes or be located on separate chromosomes that often display presence/absence variations within a population, known as conditionally dispensable or accessory chromosomes [4,6]. The common occurrence of these bipartite genomes led to the “two-speed” model for pathogen genome evolution [4], suggesting that specific regions form sites of rapid genomic diversification to facilitate coevolution during host interactions [1,4,5].

### Transposable elements shape “two-speed” genomes

**Fig 1:**
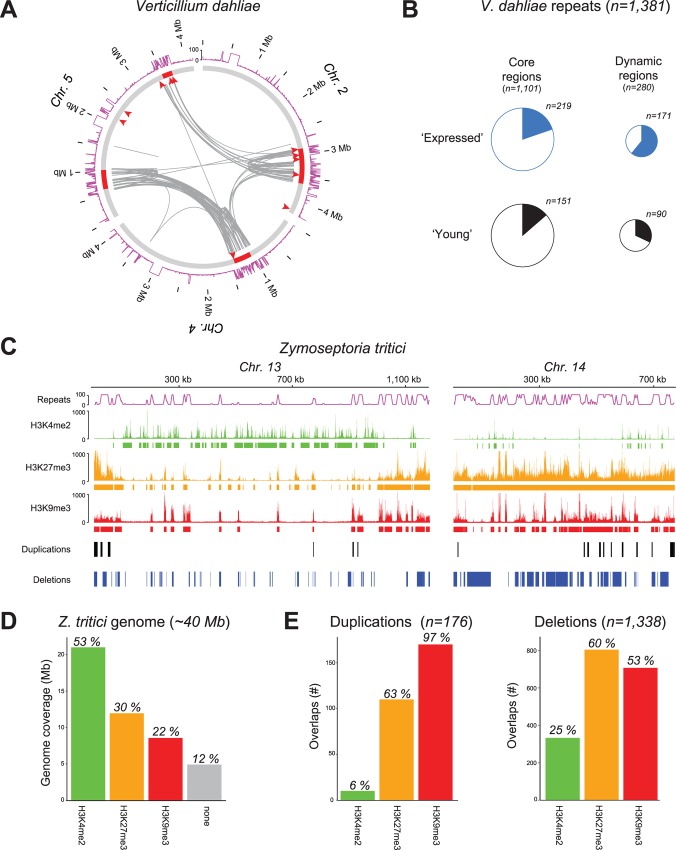
Dynamic genomic regions are associated with transposons and with distinct chromatin landscapes. (A) In *V*. *dahliae* strain JR2, repeat-rich dynamic effector regions that evolve by genome rearrangements (indicated by red arrow heads) and by extensive segmental duplications (links between highly similar duplicated regions shown in grey) are located on chromosomes 2, 4, and 5 (red highlights). Repeat density along the chromosomes is indicated by a pink line (summarized as percent nucleotides in genomic windows of 5 kb, with a slide of 0.5 kb). (B) Dynamic genomic regions in *V*. *dahliae* are enriched in transcriptionally “active” and evolutionary “young” repetitive elements when compared with the core genome [7]. (C) Different histone modifications can be associated with core (Chr 13) and conditionally dispensable (Chr 14) chromosomes in the wheat pathogen *Zymoseptoria tritici* (as previously reported [13]). Repeat density along the chromosomes is indicated by a pink line (summarized as percent nucleotide in genomic windows of 5 kb, with a slide of 0.5 kb). Publicly available chromatin immunoprecipitation sequencing (ChIP-seq) samples [13] were mapped to the *Z*. *tritici* genome, and enriched regions were subsequently identified using RSEG [28]. DNA associated with euchromatic (H3K4me2, green) and heterochromatic (H3K27me3, orange; H3K9me3, red) marks are indicated, and significantly enriched genomic regions are marked with a solid line. Structural variations (duplications, black; deletions, blue) were identified by CNVnator, using publicly available resequencing data of multiple *Z*. *tritici* strains [29]. (D) The number of nucleotides (in Mb) of the *Z*. *tritici* genome covered by different histone regions (defined by RSEG) for euchromatin (green) and heterochromatin (orange, red) are shown by bar charts. (E) The number of duplications and deletions overlapping with histone regions (see above) are shown by bar charts.

It is generally observed that the dynamic regions of two-speed genomes are enriched in TEs, yet it remains undemonstrated how they mechanistically contribute to genome variability [1,4,5]. Recently, the contribution of TEs towards the evolution of the two-speed genome in the vascular wilt pathogen *Verticillium dahliae* was reported [7]. Extensive genome rearrangements are generated by double-strand repair pathways that erroneously utilize stretches of homologous sequence at an unlinked locus, the majority of which occur around TEs simply as a consequence of their abundance and sequence similarity (Fig 1A) [7]. Genomic rearrangements often occur at dynamic regions that are enriched for recent segmental duplications, generating genetic material subject to evolutionary diversification, e.g., by reciprocal gene loss (Fig 1A) [7,8]. Furthermore, these dynamic regions are enriched in in planta induced effectors [8] and evolutionary young and “active” TEs (Fig 1B). These TEs likely contribute to accelerated genomic diversification of dynamic effector regions [7].

### Chromatin biology impacts the adaptive evolution of filamentous plant pathogens

Chromatin, a complex of nucleic acids and proteins, determines the physical shape and organization of DNA within the nucleus [9]. In many eukaryotes, highly repetitive regions are composed of tightly condensed chromatin, referred to as heterochromatin, as opposed to open chromatin or euchromatin. Heterochromatin functions to silence repetitive and neighboring DNA and to repress recombination in many eukaryotic genomes [10]. However, these observations are inconsistent with data from fungal plant pathogens since repeat-rich regions of the two-speed genome are enriched in structural variations [7,11], and genes located in these regions often show highly coordinated expression [4]. To address this conundrum, and to understand the formation, maintenance, and transcriptional regulation of bipartite genomes, it is necessary to analyze genome structure and organization through the study of chromatin biology.

### Chromatin and genome organization

**Fig 2:**
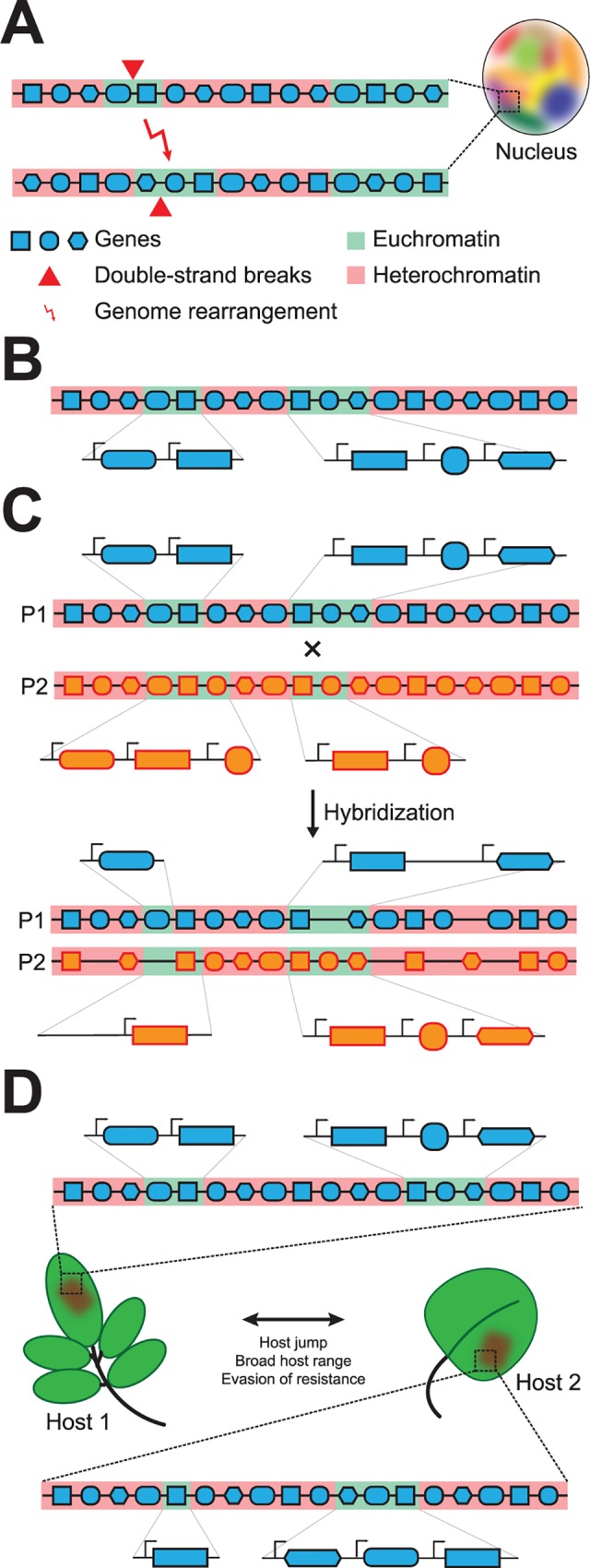
Impact of chromatin organization on adaptive (genome) evolution. (A) Genome rearrangements (red arrows) occur in open chromatin regions (euchromatin, green background; heterochromatin, red background) that are in spatial proximity within the nucleus. Chromosomes are shown as lines and genes as differentially shaped symbols. Spatial organization of the nucleus is highlighted by nuclear regions that are occupied by distinct chromosomes (different colors). (B) Chromatin influences gene expression, as genes located in open chromatin are transcribed (arrows), while genes located in heterochromatin are transcriptionally silent. (C) Interspecific genome hybridization leads to genome restructuring, often accompanied by extensive gene loss, and changes in transcriptional profiles, which can be influenced by differences in chromatin between parental species (genes from two parental lineages are indicated by orange and blue, respectively). (D) Host jumps, specialization towards a specific host, and evasion of host immunity can be influenced by changes in chromatin that translate to alterations in gene expression or DNA content.

Chromatin features such as accessibility, histone modifications, and chromosome conformation contribute to the organization of a genome. Recent modeling of mammalian and yeast genomes suggests that genomic rearrangements can be accurately predicted based on two chromatin features alone; chromatin openness and DNA contact in the nucleus (Fig 2A) [12]. Chromatin studies in most fungi remain scarce, but recent work on the fungal wheat pathogen *Z*. *tritici* shows marked differences in histone modifications between core and conditionally dispensable chromosomes (Fig 1C) [13]. Only half of the genome is occupied by histones carrying modifications that are commonly associated with heterochromatic regions, such as histone methylation of lysine residues on histone 3 at position 9 or 27 (H3K9me3 or H3K27me3). Notably, the majority of the detected structural variations colocalize with these heterochromatic regions (Fig 1C–1E). This suggests a further link between chromatin and genome structure, and despite the general thought that heterochromatin suppresses genomic variation, heterochromatic regions appear highly variable in the plant pathogenic fungi analyzed to date (see [6] for additional examples).

A possible mechanism to explain the link between chromatin and genome structure is that heterochromatic regions are more prone to DNA breaks during replication that, when repaired, could result in structural variations [1,14]. In *Neurospora crassa*, heterochromatin is highly enriched for a specific modification of histone 2, γH2A (γH2A.X in mammals), which generally marks double-strand DNA breaks [15]. It is not known why this histone modification marks these regions, but if it follows its canonical role, these regions showing elevated rates of DNA breaks would be prone to erroneous replication-based repair. Additionally, *N*. *crassa* chromosome conformation maps indicate frequent and strong contact between heterochromatic regions [16]. Taken together, the increased rates of genomic variation at heterochromatic regions may arise from DNA damage during the replication of heterochromatic DNA, followed by error-prone replication-based repair or template switching between contacting loci.

### Chromatin and gene expression

The majority of chromatin studies using plant-pathogenic fungi have focused on their transcriptional impacts (Fig 2B) [17–19]. Canonical repressive chromatin marks, such as H3K9me2/3 or H3K27me2/3, are significantly enriched at regions known to harbor genes important for pathogenicity [17,18]. Additionally, the canonical activating mark H3K4me2/3 was shown to play a significant role in regulating gene expression and promoting growth and virulence in *Magnaporthe oryza*e [19]. Collectively, these studies indicate that deregulating chromatin significantly impacts fungal transcription, growth, and development. To gain further understanding into the regulation of pathogenicity, it is necessary to determine the key enzymes responsible for reading, writing, and erasing these epigenetic modifications.

### Chromatin and adaptation

Chromatin modifications can functionally diversify between species. For example, the addition of methyl groups to histone 3 at lysine 36 (H3K36me3) functions in guiding both DNA mismatch repair and messenger RNA (mRNA) splicing machinery in humans [20,21], while in the yeast *Saccharomyces cerevisiae*, the same mark functions to suppress intragenic transcripts [22]. Thus, although the overall localization of the mark at transcribed genes is conserved, species evolved to utilize the mark differently. Along with functional diversification between species, chromatin architecture can vary within a population [23]. It has been proposed that environmental conditions can result in heritable epigenetic variation and act as a substrate for so-called “Neo-Darwin” selection [24].

Given that chromatin regulation can evolve, is variable within populations, and can be environmentally influenced, it can be anticipated that chromatin affects host–microbe interactions. For example, several documented cases show that plant pathogens may undergo, or are the result of, interspecific genome-hybridization events [25]. These events are accompanied by complex gene expression changes, likely influenced by parental chromatin structure, and by genome reorganization in the hybrids (Fig 2C). These changes in expression and DNA content can lead to alterations in the hybrid’s ability to compete for a particular niche and could facilitate a host jump (Fig 2D). Pathogens that are well adapted to a specific host may also undergo chromatin-based regulatory changes that alter their host interaction. For example, expression of the *Avr3a* effector in *Phytophthora sojae* can change between generations, allowing for evasion of recognition by the corresponding host immune receptor Rps3a in soybean [26]. The mechanism for this switch has not been reported, but experimental evidence shows an enrichment of small RNA (sRNA) at the locus in silenced versus *Avr3a*-expressing lines with no genetic mutations reported, suggesting epigenetic regulation (Fig 2D). The prevalence of such an epigenetic evasion strategy in other plant–microbe interactions remains unknown. It is also interesting to speculate that variation in chromatin structure within a pathogen population could influence a broad-host-range pathogen to form strains with increased fitness on a particular host. Conceptually, this could be achieved through alterations in the chromatin-based regulation for the optimal timing, rate, and order of transcriptional events leading to successful infection. That is, alterations in transcription to dampen or augment an infection strategy better suited for one host versus another could influence the evolution of a plant–microbe relationship (Fig 2D). Future experiments to address these possibilities may uncover yet additional layers influencing the evolution of plant–microbe interactions.

### Outlook

Chromatin biology can impact filamentous pathogens across spatial and temporal scales, from governing genome organization to controlling the expression of its individual parts. We conceive that complex chromatin structures in pathogenic fungi will influence not only coordinated effector expression in dynamic genomic regions but also structural variations, thereby further linking genome and chromatin structure to genome evolution and adaptation. Additionally, chromatin structure also plays crucial regulatory roles in establishing symbiotic interaction between the fungus *Epichloë festucae* and its plant host [27]. Thus, studying the impact of chromatin biology on genome organization can broaden our knowledge and potentially provide mechanistic understanding of the evolution of two-speed genomes in plant pathogens and, in general, of adaptive genome evolution in plant–fungus interactions.
